# Comparing extended and standard lymphadenectomy surgeries for bladder cancer (complications, final outcomes and effects of neoadjuvant chemotherapy)

**DOI:** 10.1186/1753-6561-9-S1-A37

**Published:** 2015-01-14

**Authors:** GS Thiara, P Black

**Affiliations:** 1 Royal College of Surgeons in Ireland, Dublin 2, Ireland; 2 Department of Urology, Vancouver General Hospital, Vancouver, BC, Canada

## Background

Bladder cancer is a common urologic cancer and the 6th most common cancer in Canada. In terms of malignancy, this cancer has the highest recurrence rate. The most common type of bladder cancer is Transitional Cell Carcinoma (TCC). Worldwide, each year, bladder cancer is diagnosed in 275,000 people. In the past 10 years, bladder cancer accounted for 7.5% of all detected cancers in men and 1.8% of those of women in Canada. More than 2100 deaths per year occur in Canada as a result of bladder Cancer. Last year, a study at the Vanderbilt University Medical Center in Nashville illustrated that extended lymphadenectomy consistently provided an advantage over standard approaches at the time of cystectomy with insignificant increases in morbidity. We had similar questions and decided to do a study to build on current knowledge. In this report, I am comparing extended and standard approaches to lymph node removal with respect to complication risks, patient survival outcomes and neoadjuvant chemotherapy responses.

## Methods

In this study, patients were matched by basic information (age, BMI, ASA) and pathology characteristics. 75 patients were compared in terms of complications. We divided these complications into in-hospital (2 weeks or less), early (2 weeks to 90 days), and late (greater than 90 days). 130 patients were compared in terms of recurrence rates and final outcomes: alive with disease, dead of disease, dead of other cause or no evidence of disease. Furthermore, analysis was done on patients who received neoadjuvant chemotherapy and looked at their final outcomes and recurrence rates as well.

## Results

Complications were higher in early and late categories in the standard type of surgery. However, in-hospital complications were higher in extended lymphadenectomy. Final outcomes showed increased rates of no evidence of disease in extended lymphadenectomy and lower recurrence rates. Patients who had neoadjuvant chemotherapy also showed reduced recurrence rates and more elimination of pathology in the extended group.

## Conclusions

Extended lymph node removal (ELNR) illustrates reduced early and significantly reduced late complication rates. However, early complications are higher possibly due to the increased invasiveness of the procedure. ELNR also demonstrates reduced recurrence rates and more favorable survival outcomes than the standard type of surgery. The same positive findings are also seen in patients who had neoadjuvant chemotherapy.

